# Shared Decision Making in an Integrated Mental Health and Vocational Rehabilitation Intervention: Stakeholder Practices and Experiences

**DOI:** 10.5334/ijic.5509

**Published:** 2020-11-30

**Authors:** Kathrine Hoffmann Pii, Lisbeth Hybholt, Rie Mandrup Poulsen, Lene Falgaard Eplov, Mathias Meijer

**Affiliations:** 1 University College Copenhagen, Institute of Nursing and Nutrition, Copenhagen, DK; 2 Centre for Relationships and De-escalation, Mental Health Services Region Zealand, Slagelse, DK; 3 Mental Health Services East, Psychiatry Region Zealand, Roskilde, DK; 4 Copenhagen Research Center for Mental Health – CORE, Rehabilitation, Recovery and Shared Care, Mental Health Center Copenhagen, Mental Health Services in Capital Region of Denmark, Hellerup, DK

**Keywords:** shared decision making, integrated care, mental health care, vocational rehabilitation, common mental disorders

## Abstract

**Introduction:**

A Danish integrated mental health care and vocational intervention was developed to support the return-to-work process for people with common mental disorders. Shared decision making was a core element of the intervention to ensure a person-centred approach. The study aim is to describe how shared decision making was practiced and experienced and to discuss its potential in this integrated care context.

**Theory and methods:**

Shared decision making practice and experience was studied in participant observation (n = 20), interviews (n = 12), focus groups interviews (n = 2), and shared plan documents (n = 12). Research methods and analyses were guided by theoretically defined ideals of shared decision making.

**Results:**

Shared decision making constituted a general value rather than a structured method in practice. Clients experienced a more person-centred collaboration with professionals, compared to the regular vocational system. Contextual factors regarding vocational legislation and the intervention design influenced the decision latitude.

**Conclusion:**

Shared decision making has the potential to support a person-centred approach in integrated services. However, we recommend clarifying decisions applicable for shared decision making, to ensure thorough training, develop and test decision aids, and ensure supportive organisational conditions for shared decision making in interprofessional collaboration.

## Introduction

Common mental disorders such as depression, anxiety and stress disorders are frequent causes of sick leave in high-income countries, including Denmark [1,2,3,4]. In Denmark, common mental disorders are the main reason for early retirement [2,5], and anxiety and depression alone account for annual production losses of 1.6 billion Euros (2010–2012 price levels) caused by absence from the labour market due to sick days, early retirement benefits and early death [2]. People with common mental disorders who want to keep, return to or find a job often have a combination of social problems and health issues that call for integration of public services but are often met with fragmented and uncoordinated services [6]. Research has shown that adequate mental health care cannot stand alone when supporting people in returning to work [4]. Integration of health care and vocational services has shown to improve return to work for people with severe mental disorders [7,8]. Only little evidence exists regarding the effectiveness or feasibility of intersectoral integration efforts for persons with common mental disorders [1].

However, integrating mental health care and vocational rehabilitation has proven to be difficult [9,10,11]. Research has identified cooperation between multidisciplinary stakeholders across sectors as one of the main barriers [6,12,13]. A meta-synthesis of client experiences with return-to-work interventions showed that the lack of coordination between public services left clients confused and uncertain about how and when to return to work. It was suggested that return-to-work processes should occur in collaboration between clients and professionals [6]. It was also recommended to make the client perspective the central organising principle and that professionals should provide services that are respectful of and responsive to the client’s individual preferences, needs, and values and let client values guide decisions [6, 13,14,15,16,17,18,19].

IBBIS, a Danish intervention, integrated mental health care and vocational intervention to support persons on sick leave due to common mental disorders (for more information see study protocols [20,21]). This was among others built on principles from IPS [14] and the SHARP-at work intervention [22].

IBBIS incorporated shared decision making to support a person-centred approach and clients’ active involvement in decision making. Shared decision making is an interactive process in which stakeholders share information about available options and their potential benefits and risks and discuss preferences in order to arrive at a decision that they can agree on [23]. Shared decision making has primarily been used in health care and was originally developed to support physician-patient collaboration and decision making related to somatic illness, but its relevance has been tested and evaluated in various fields including mental health care [24]. Its potential in integrated health and vocational rehabilitation has rarely been explored [16,17].

The contextual conditions for using shared decision making in an integrated mental health care and vocational setting differ from the clinical setting in which it was developed. The aim of this report is to describe how clients and multidisciplinary teams practiced and experienced shared decision making and to discuss its potential in integrated mental health care and vocational rehabilitation.

## Theory and Method

### Study context

The IBBIS integrated intervention was developed by researchers from the Mental Health Center, Copenhagen. The intervention consisted of mental health care and vocational rehabilitation which was offered to sick-leave clients with common mental disorders [20,21] as part of the comprehensive social security system in Denmark. The IBBIS intervention was launched and tested as part of a large reform of the Danish sickness benefit legislation which aimed to fasten provision of support and thereby return to work rates [25]. The intervention was delivered in four Danish capital municipalities by two multidisciplinary teams consisting of employment specialists, care managers, a psychiatrist and a psychologist [20,21] (study results regarding the intersectoral collaboration is reported in [26]).

Care managers delivered mental health care as a standardised stepped care program, where clients were offered the treatment that was most effective and least invasive and resource-intensive based on initial assessment by a care manager, the psychiatrist or the psychologist. Treatment was provided according to diagnosis-specific care plans, e.g., individual psychoeducation, cognitive behavioural therapy, stress reduction coaching or group-based mindfulness stress reduction course with regular monitoring and assessment. Employment specialists provided vocational rehabilitation that included vocational assessment of work capacity, a vocational plan, support in returning to the existing workplace, job search support, case management and coordination with other public social services. Care managers and employment specialists had primary interactions with clients. Care managers had a background in nursing, occupational/physiotherapy or social work and were employed full time. Employment specialists included social workers or other professions with work experience in municipality vocational rehabilitation. Some employment specialists worked part time for the regular municipality vocational rehabilitation and part time in IBBIS. Multidisciplinary integration of teams was supported by co-location of team members to ensure communication and collaboration, and the teams received monthly multidisciplinary supervision.

After mental health assessment, each client met with a care manager to formulate a mental health care plan. Subsequently, the client met with an employment specialist to formulate a vocational rehabilitation plan. A roundtable meeting followed, during which client, care manager and employment specialist jointly made a shared plan specifying the necessesary steps to facilitate a return-to-work process that was tailored to the client’s mental health, work situation and everyday life and aligned with vocational legislation and the IBBIS intervention design. The formulation of the shared plan and potential adjustments required a shared decision making process among the three stakeholders. In IBBIS, shared decision making was used as a principle to guide collaboration between the client and professionals. The intervention manual defined shared decision making as a process involving the client and multidisciplinary team as active participants in decision making in which all stakeholders share information and preferences and agree on decisions. In addition to the intervention manual, professionals received one days training in the principals of shared decision making.

### Theoretical framework: Shared Decision Making

Shared decision making has been defined in several ways since its onset in the early 1990s but central values and understandings are shared across the differences [23]. The theoretical underpinnings of this study is found in the three-talk model developed and revised by Elwyn et al. [27,28]. The model depicts three stages that support the deliberation process of shared decision making: team talk, option talk and choice talk [28]. The first stage specifies that a decision is to be made and that the client’s opinion matters. The second stage discusses possible options and the pros and cons of each option. The last stage focuses on reaching a shared decision that reflects the informed preferences of the client. Different tools have been developed to measure the quality of shared decision making, eg. OPTION 12 [29], OPTION 5 [30] observation tools and SDM-Q-9/SDM-Q-9-DOC evaluation questionnaires for patients and doctors [31]. In this study, we used the OPTION 12 observation measure [29], which operationalises the three-talk model into 12 elaborated items to guide observations.

### Methods

The study was based on a qualitative multi-method research design including participant-observations of roundtable meetings with clients and professionals (n = 20), follow-up interviews with clients (n = 12) and focus group interviews with the multidisciplinary IBBIS teams (n = 2). Shared plan documents (n = 12) were also included as data. Data was generated over a period of 13 months from December 2016-January 2018, eight month after the onset of the intervention.

Characteristics of client study participants are reported in Table 1.

**Table 1:** Client study participants.

Client	Diagnosis	Team	Observation	Interview	Gender

1	Stress	1	X		F
2	Stress	1	X		F
3	Stress	1	X	X	F
4	Depression	1	X	X	F
5	Depression	1	X		M
6	Depression	1	X		F
7	Stress	1	X	X	F
8	Depression	1	X		M
9	Stress	1	X	X	F
10	Anxiety	1	X	X	F
11	Stress	1	X	X	F
12	Stress	2	X	X	F
13	Stress	2	X	X	F
14	Anxiety/depression	2	X		F
15	Anxiety	2	X		F
16	Stress	2	X		M
17	Stress	2	X	X	F
18	Depression	2	X	X	F
19	Stress	2	X	X	M
20	Stress	2	X	X	F

The recruitment of the clients was initiated by professionals, who excluded clients they assessed being too vulnerable for participating. After this initial procedure, clients were informed orally about the purpose by the observing researchers (LH and KHP) and received written information before consenting.

Roundtable meetings were chosen as observation site because of their centrality in establishing the integrated approach in IBBIS by initiating stakeholder collaboration and formulating the shared plan for the client. The meetings necessitated making several important decisions and were thus an ideal situation to observe the decision-making process. The aim was to record actions in their naturalistic surroundings focusing on the conversation content and the dynamics of interactions and discussions. Observers (LH and KHP) made field notes during and after meetings, which lasted 30–60 minutes, to capture descriptive details of interactions and verbatim conversations [32]. Observations were overall guided by the three-talk model, observing the framing of the meeting, the decisions discussed and made, and the dynamics of collaboration between the three stakeholders. The shared plans formulated during and after meetings were included as supplemental data.

KHP and LH made independent observations and talked with stakeholders before and after the meeting. After the observations, clients were asked to participate in interviews. Twelve of 20 clients consented to interviews.

Interviews were conducted by LH and KHP by telephone and focused on client experiences of the roundtable meetings and involvement in the decision-making process. The interviews were conducted 1–3 days after meetings and lasted 13–34 minutes (mean, 25 minutes). The interview guide included questions about preparation for making decisions, options discussed, experience of options’ pros and cons and client experience of influencing decisions. These questions were guided by the three-talk model [28].

Two focus group interviews with six and eight professionals from multidisciplinary teams were conducted by KHP to investigate their perceptions of and experience with shared decision making and the organisational conditions of working with shared decision making. Focus group interviews lasted 90 minutes.

### Analysis

The analysis combined deductive and inductive approaches. Each data set was first analysed separately, and findings were compared across data. The decision-making process was analyzed deductively using the OPTION 12 measurement to identify practices in line with the core elements of the shared decision-making process [29]. LH and KHP analyzed the field notes separately and compared their identification and categorization of practices. In case of disagreement, it was discussed how the observed practice should be categorized according to the shared decision making ideals described in OPTION 12 to qualify the analysis. Furthermore, KHP identified numbers and types of decisions discussed to gain an understanding of the decision latitude at roundtable meetings. Data from shared plan documents were included in the analysis of the decision-making process. Analysis of interviews and focus group discussions was inductive, focusing on stakeholders’ experiences of practicing shared decision making, e.g. how they interpreted the ideals of shared decision making and how it linked to professional ideals and organizational conditions for practicing shared decision making.

### Ethical considerations

The study was conducted in accordance with the Declaration of Helsinki II Principles. Informed consent was obtained from all stakeholders before observations and interviews. Interviews were conducted by telephone rather than face-to-face to minimise potential stress related to sharing experiences with a stranger for clients in a vulnerable mental state. To ensure clients’ wellbeing, professionals assessed whether they were too vulnerable for study inclusion. To ensure participants’ anonymity, pseudonyms are used and identifying details were excluded or changed. Data was handled according to The General Data Protection Regulation (GDPR) (REGULATION (EU) 2016/679) and The Data Protection Act (Act No. 502 of 23 May 2018, Denmark).

## Results

We present the types of decisions made at roundtable meetings, the results according to the three-talk model [28], and contextual factors influencing the practice of shared decision making.

### Decision types at roundtable meetings

During roundtable meetings, a broad range of decisions were discussed and made. Between 10 and 25 decisions were discussed at each meeting. Table 2 presents a decision typology with examples of content and options. Most decisions related to the IBBIS content, in terms of planning concrete activities in the course. Other decision types were the return-to work process, collaboration between stakeholders and goals and goal setting. Although goal setting was central to the shared plan, other issues and decisions dominated discussions. Discussions did not necessarily lead to actual decisions at the meeting, such as a concrete return-to-work date. Furthermore, professionals emphasized that the shared plan was provisional and could be adjusted.

**Table 2:** Typology of decisions discussed at the roundtable meetings.

Decision types	Content	Examples of options discussed

**The IBBIS content**	The individual focus in the IBBIS course	reducing stress increasing activity gaining confidence to seek new job
	Activities in the intervention	writing a stress diary participating in mindfulness-based stress reduction course writing a CV defining dream job discussing employment with employer and employment specialist
	Planning IBBIS meetings	suitable weekdays/time for meetings meeting frequency type of meeting with employment specialist (telephone or face-to-face) client’s holidays
**Return-to-work process**	Job function and assignments	alternative work assignments reduction in workload physical workplace adjustments new colleagues
	Estimated fitness for duty (termination of sick leave benefit)	set a concrete date postpone date decision
	Realistic date for return to work	set a concrete date postpone date decision
	Stepped return-to work plan	work hours progression per day and week
**Collaboration between stakeholders**	Client role and responsibility	specifying homework activities or issues to investigate
	Roles and responsibilities among professionals	contacting other stakeholders investigating different issues related to health care or vocational legislation
	Collaboration with employer and colleagues	how and what to communicate to the workplace
	Collaboration with other stakeholders	how to collaborate with other health professionals, the municipality or the union
	Involvement of family and friends	inviting spouse to IBBIS meetings how to include family and friends in recovery
**Goals and goal setting**	Long term goals	better work-life balance improve self-esteem find a job or change job cope better in current job
	Temporary goals	get out of bed every day clean up at home walk the dog go on social visits
**Excluded decisions**	Client requests that exceeded the IBBIS framework	Supplemental psychological therapy Additional sessions with care managers

Professionals dismissed some issues that clients raised. These included desires for supplemental psychological therapy outside IBBIS, extension of their inclusion in the IBBIS trial, more therapeutic sessions with care managers or issues that were defined by the legislation, for example expanding the length of benefit.

Formulating the shared plan, which summarised the mental care plan and vocational plan into specific goals for the individual client, was central to the roundtable meeting. Although the overall goal of returning to work was predefined by the intervention, sub-goals were often developed to fit the client’s situation and abilities to return to work, such as a trainee course or a stepped return-to-work course. Vocational rehabilitation could be postponed if the client was too ill and needed to be “protected from the vocational system”, as one care manager expressed it. In such cases, health-related goals were prioritized and vocational goal decisions were postponed, as described in a shared plan:

“Client A needs, via an integrated IBBIS-service, to reclaim a solid foundation in her life, which at present is very fragile. She needs to gradually experience that her strength returns both in terms of coping and involvement in relationships with other people and living an outgoing lifestyle, which she is motivated for and is able to accomplish in her family network. When she feels that some of her strength is reestablished, a job seeking process will begin. She has never had any difficulty finding a job. There might be a need for support this time through the municipality specialist consultant. The feeling of security and clear boundaries in the job is more important that the work content, she has expressed.” (Shared plan, client A)

The formulation of goals in the shared plan varied. Whereas the above example specifies an individually defined goal, other examples were more general, e.g., “Clients B can return to work at the workplace on full-time terms” (Shared plan, client B). Other goals reflected integrated mental health and vocational goals, e.g., “Client C needs to reenter the job market as quickly as possible. This will lead to a better economy, work identity and reduce loneliness.” (Shared plan, client C).

### Team talk

#### Framing equal collaboration

A central premise in shared decision making is establishing an equal working relationship among stakeholders. In the focus group interviews, IBBIS professionals highlighted the importance of “equality” and “reaching consensus” without anybody “feeling forced”. They stressed that an equal collaboration first and foremost required transparency in exchanging knowledge among stakeholders. This would create a common ground for making the best plan and the best decisions in collaboration with the client. At meetings, the roles of each stakeholder were explicated; one care manager said:

“The employment specialist is an expert on vocational rehabilitation, I am on health-related issues and you are the expert in your life”.

During roundtable meetings and in focus group interviews, the client’s role was described as “being at the center”, “being at the helm”, “being the expert of their own life”, “being in charge”, “being the main character”. Their role in formulating the shared plan was also stressed during meeting:

**Table:** 

Employment specialist:	“The most important thing is that the three of us are here to make a shared plan that suits you. You are the main character. I will present a short summary of what you and I have worked with and the same goes for the care manager”.
Care manager:	“And you will of course add to that. We need to ensure that we are following the same path”.
Employment specialist:	“Yes we are not on two paths here, it will be integrated – that is the whole purpose (smiles). We’ll end the meeting by formulating a shared plan, which we’ll send to you and you can comment on it”.
Care manager:	“We are following the same path – you are at the helm, so you gain what you wish”.
(Observations, KHP)	

Professionals made explicit efforts to neutralize and minimize power imbalances between stakeholders and often described the IBBIS approach as a contrast to the conventional vocational system in municipalities. Clients expressed that they experienced a more equal relationship with the IBBIS professionals, compared to the conventional vocational system.

“They didn’t push me, nobody was rude. I felt I could say what I felt”. (Client D)

Professionals explained clients that they had not shared information with each other before the meeting. The purpose was to create transparent knowledge exchange and dismantle professional alliances. Clients reported that it was nice to know that the professionals had not talked behind their backs. In general, clients described professionals as providing joint support. They felt that the professionals supported them with expertise from their respective fields, which created a good overall solution:

“I have that feeling, that somebody is holding my hand and that is so important. First, [care manager] helped me the first few times and then [employment specialist] took the other hand and somehow it became a holistic solution”. (Client A)

Despite the ideal of equality, professionals primarily led the meetings. Clients often displayed hesitation in meeting and contributed when directly invited. When asked to reflect upon this dynamic of roundtable meetings, professionals said that they could improve client involvement by encouraging them to say more and empowering them to take more control. However, some clients said they were uncomfortable taking the lead because of low self-confidence related to their mental condition. One client said: “I wouldn’t be able to take the lead, I often suppress my problems”. (Client D)

Others expressed feeling generally insecure and powerless towards the vocational system due to prior bad experiences with ‘the system’; as one said, “I felt left behind. I started [the sick leave benefit case] and I had these fights with the sickness benefit system and felt all alone in this ‘land’”. (Client E)

#### Framing decision making

The framing of decisions and the decision-making process at meetings was less clear. Most clients expressed that they had been unsure about the purpose of the meeting beforehand, leading to feelings of stress and insecurity.

“I was actually a bit scared that we had to decide on a concrete date (for return to work). So that was in the back of my mind and I experienced symptoms of stress. My heart was beating rapidly as I was waiting. I thought, what if I am told that I have to return to work in two weeks or on Monday next week or something. I didn’t know what they expected from me”. (Client F)

These feelings often dissipated during the meeting, and clients experienced the atmosphere as becoming more relaxed. They experienced their perspectives as being heard and respected and the purpose of the meeting became clearer to them as it continued.

The shared plan was often used to structure the conversation at the meeting, summarise discussions and translate discussions into concrete goals and plans. The shared plan thus represented decisions discussed and, ideally, agreed upon at the meeting. However, most clients did not perceive the roundtable meeting as a decision-making meeting or that decisions were being made. We noted that decision making was rarely explicated in the framing of the meeting, despite the fact that many decisions were made.

The professionals perceived shared decision making as a value rather than a structured methodology, and they explained that they had not received much training in shared decision making. Their knowledge about the approach was primarily from the intervention manual, but care managers also saw some similarities between shared decision making and their usual therapeutic approaches.

### Option talk

#### Sharing knowledge

The objective of option talk is to share knowledge, clarify relevant options and reflect on their pros and cons in order to make a decision. The professionals described care managers as sharing knowledge about mental health care, typical symptoms and responses of illnesses and therapeutic approaches and employment specialists as sharing knowledge about legislation, job search and return-to-work processes. Clients shared knowledge about personal values, preferences, family life and employment history. Overlaps sometimes occurred between professionals’ knowledge, e.g., return-to-work processes and mental illness symptoms. Professionals described sharing knowledge as also about getting to know each other, gathering all relevant information to integrate services meaningfully and reaching a common ground for the return-to-work plan. As demonstrated in the decision typology, meetings often centered on discussions and decisions about the IBBIS content. Decisions were based on a shared assessment of the clients’ condition, motivation and resources. In the following example, a client shares her experience of the treatment plan in her everyday context and a care manager shares her expertise on recovery processes.

**Table:** 

Client:	(crying and upset) “I need to say that I have a very very bad day, I am getting worse by attending this (IBBIS). I need to find out if we think this is good for me, I can’t manage it at home, there is so much and nobody to help me, it is so demanding. I think it is good for me, but I can’t manage it all, the exercises, I can’t. So, we have to call it off. Then I’ll just have to find an easy job and shut it all down – we have to find out what is best for me”.
Care manager:	“We can adjust the exercises; it’s not meant to stress you out. Let’s talk about it on Thursday when the two of us meet”.
Client:	“I just have this huge time pressure on me at home and it takes up all my energy and now I don’t have a car anymore and need to take a bus in the morning and it is such a pressure, we have not settled at all with that routine and I can’t sort out things, I don’t have any orientation”.
Care manager:	“We can adjust the exercises, it is very common that people at your stage, feel that everything adds to the pressure, it sounds like you are under a lot of pressure right now”.
(Observation LH)	

As the example illustrates, the exchange of knowledge between client and care manager contributed to adjusting the treatment.

Sometimes clients were hesitant to share knowledge. Some were suspicious about hidden agendas and others felt vulnerable when sharing knowledge. A client worried about the way she had portrayed herself at the meeting and was unsure of the consequences:

“… when [the employment specialist] said ‘You have done so many different things in your career’, then I got a bit too smart and said I could do this and that, I have done this and that. BUT there is a reason why I can’t work as a [X] anymore. I forgot to say that, but I felt time was running out and that I shouldn’t take up all this time. I had to be careful not getting too smart, but at the same time, be careful that I didn’t say too much. I was afraid that I would lose my credibility…as if I was not willing [to work], but just making up excuses for not returning to my old profession. That was what I feared, I don’t know why. But afterwards, I thought why the hell didn’t I say, ‘I can do many things, but if you ask me to work in my old profession, then I will crumble’. But I don’t want to be that kind of person who whines and tries to make up poor excuses why I can’t do this or that”. (Client A)

In some cases, clients’ mental conditions and uncertainty about their capabilities, preferences and values made it challenging for them to share knowledge to facilitate discussing options.

#### Explicating options

Despite the many decisions made during the roundtable meetings, options and their pros and cons were often unclear. Visual decision aids that support a structured shared decision-making process were not used to guide the discussion of option pros and cons. Although options and their pros and cons were sometimes defined and constructed during the conversation, clients most often experienced options as unclear or unavailable.

“It was like, ‘we’ll continue the way we are doing this’. I felt that [the employment specialist] expected that I could say if I was ready or not to return. I was a bit left to myself. Of course, they shouldn’t have to make that statement on my behalf. But they could perhaps have shared their thoughts and suggested what to try. But no, I didn’t feel I had a choice. I was told that we’ll continue as planned”. (Client E)

### Decision talk

During decision talk, preference-based decisions should be made and reflect what matters most to the client. While most clients experienced the content of the roundtable conversations as relevant and meaningful, they did not necessarily experience decisions as being made, despite the fact that the shared plan was intended to reach agreement on shared goals. As depicted in the decision typology, the decision latitude, in terms of the range of decisions discussed and the options, was broad. This reflects a high degree of flexibility and the possibility of individual adaptation. Often, professionals also stressed that goals and plans were temporary and could be adjusted during the course. This flexibility reflects the values of shared decision making.

However, both the IBBIS design and vocational legislation limited the flexibility and range of individual adaptions. For instance, only a fixed number of sessions was possible in IBBIS although some clients wanted more sessions. Also, the Danish legislation defined the maximum length of sickness benefit.

### Contextual factors challenging shared decision making in integrated vocational rehabilitation

A range of contextual factors influenced the practice of shared decision making in IBBIS. First, integrated vocational rehabilitation involves multiple actors, professional fields and institutions. Decision making in this context is a multifaceted effort that is conditioned by different values, aims and limitations. In the IBBIS intervention, the decision latitude was restricted by the vocational legislation and the intervention design. The values of the involved stakeholders and institutions also influenced the decision-making process. Despite the intervention’s integrated approach, the institutional aims and values of mental health care and vocational rehabilitation sometimes conflicted. Professionals expressed these conflicting aims and values as being prominent at the onset of the intervention but decreasing as they got to know each other’s professional fields and competencies through a collegial relationship. Co-location and daily collaboration supported the integration and reduced internal professional conflicts. Professionals described employment specialists’ part-time employment as problematic because it prolonged the establishment of integrated collaboration and tied employment specialists to the aims and values of the conventional municipality vocational rehabilitation [26].

Employers from the clients’ workplace also influenced the intervention through their management of the return-to-work process. In some cases, employers did not follow shared decisions about the return-to-work process made between the interdisciplinary team and clients. In such cases, employment specialistst offered to participate in meetings between the client and employer. Integrated vocational rehabilitation context involves more actors and more institutional aims and values, increasing the complexity of shared decision making.

## Discussion

The aim of implementing shared decision making in IBBIS was to support a person-centered approach, which ultimately would improve the return-to-work process. Most clients experienced collaboration at the roundtable meeting positively and felt that their perspective was respected. This was in stark contrast to their experiences with the regular vocational system. The pressure and distrust of the vocational system and the insecurity and frustration of uncoordinated services among clients with mental illness are found in other studies [18,23,33]. Our findings show that the value of shared decision making could facilitate a better relationship between the system and the client.

The observed framing of decisions and options in IBBIS did not reflect shared decision-making theory. The broad range of decisions discussed at the meetings indicates a need to define key decisions that would benefit from a thorough shared decision-making process. Shared decision making was developed in a clinical setting and has traditionally been practiced in situations in which patients can choose between well-defined treatment opions that often have equivalent clinical effects but different implications for individuals’ daily lives. In these situations, individual preferences are crucial. In contrast, vocational legislation and the IBBIS intervention design set certain contextual limits for the decision latitude. The ultimate goal of the vocational system is return to work, and clients need to agree to this goal to receive sick-leave benefits. The mental health care plans also define the type and number of therapeutic sessions. Thus, shared decision making is relevant in this context in regard to the many intermediate goals to reach the ultimate goal of return to work.

A feasibility study of shared decision making in vocational rehabilitation confirms the importance of intermediate goals in vocational rehabilitation and the relevance of shared decision making to setting them [16]. In this study, all stakeholders identified return-to-work conditions and action plans (such as the shared plan) as the most important problems requiring shared decision making. Stakeholders agreed that shared decision making could support a realistic return-to-work plan that reflected the client’s preferences and capacities. They emphasized that these capacities should consider clients’ comorbidities. In IBBIS, the assessment of clients’ capacities included their social, economic and practical conditions, as well as comorbidities. This approach reflects the possibility of individual adaptations in the intervention. However, this flexible approach also makes it difficult to predefine options and facilitate a structured discussion of pros and cons. The feasibility of decision aids has however been questioned by Coutu et al. [16], who argue against the general recommendation in clinical settings to use such aids to support the shared decision-making process. They argue that it is difficult to develop universal aids in a vocational rehabilitation setting because options cannot be predefined and presented in decision aids; rather, they must be adapted to each case. Although this argument can be applied to the IBBIS intervention, it is worth considering that other kinds of decision aids, such as generic reflective tools and question prompts, could potentially support clients’ deliberation process and preparation for engaging in decision making at roundtable meetings.

The study showed that clients’ employers were not part of the shared decision making process at the roundtable meetings and that they sometimes became barriers for successful return to work processes. This could suggest that employers ought to be part of the shared decision making process at the roundtable meeting. However, adding employers’ presence in the decision making process could also enhance clients’ feeling of pressure and their general insecurity towards the vocational setting and thereby obstruct the establishment of equal collaboration, a point Coutu et al. also make [17].

### Implications for practice

In IBBIS, shared decision making was practiced as an overall value but was not delivered in a methodologically structured way. This gap between theory and practice is a common finding in the literature about implementing shared decision making and in many other implementation studies. Therefore, it is useful to consider structural support to improve implementation. An overview of 22 systematic reviews of shared decision making recommends that implementation include training professional communications skills, coaching patient communication skills (e.g., using question prompts) and using decision aids during the decision-making process [34]. In IBBIS, professionals received only minimal training in shared decision-making methods, and they did not use decision aids. Contrary to Coutu et al. [17], we argue that decisions aids and question prompts may help prepare clients to engage in the shared decision-making process and that their feasibility should be tested.

Furthermore, we suggest that the general insecurity experienced by clients while collaborating with the vocational system should be taken into account by enhancing their preparation for the roundtable meeting. Clarifying the meeting purpose ahead of time could reduce context-specific insecurity and stress and the use of question prompts could improve clients’ awareness of and involvement in decision-making.

As stated above, the gap between theory and practise cannot simply be explained by unwillingness on the part of professionals or patients. The realisation of shared decision making ideals is difficult due to the general challenges of changing patterns of expectations, roles, habits and routines that are institutionally embedded in the healthcare system [35]. These challenges become more complex when health care and vocational rehabilitation are integrated.

### Study limitations

The study focused on shared decision making at the first meeting between care manager, employment specialist and client, which may explain the lack of clear options and decision talk. Goal setting depends on individual clients’ situation and readiness, and their mental health conditions may hinder formulating vocational goals. We did not study shared decision making in the separate meetings between client and each professional, where decision making also may take place and may be better framed as option and decision talk. However, in focus group discussions, professionals confirmed our findings regarding the poor framing of decisions and options as they described shared decision making as a value rather than a methodology, they had been trained in.

There is an overrepresentation of clients with stress disorder. We understand this as a result of the recruitment process, where professionals excluded clients they assessed being too vulnerable to participate. The overrepresentation of women is in line with the general IBBIS population. We have not used the OPTION 12 observation tool as a systematic quantitative quality measurement as it is intended but as an analytical tool to identify practices reflecting shared decision-making ideals in our field notes.

## Conclusion

Shared decision making supported the integration of mental health care and vocational rehabilitation by facilitating a more equal collaboration between clients and professionals. However, to achieve the full potential of shared decision making, the methodology should be followed more thoroughly and be supported by training and ongoing supervision of professionals. Clients experienced a person-centred approach and their individual preferences and capacities were included at the roundtable meetings. Vocational rehabilitation legislation and the intervention design influenced the practice of shared decision making, particularly in terms of the range of decisions clients could influence. We conclude that it is essential to define decisions that are applicable for a structured decision-making process, give professionals thorough training in shared decision making, develop and test decision aids, and ensure supportive organisational conditions for interdisciplinary collaboration.
